# Personality and stress influence vision restoration and recovery in glaucoma and optic neuropathy following alternating current stimulation: implications for personalized neuromodulation and rehabilitation

**DOI:** 10.1007/s13167-020-00204-3

**Published:** 2020-04-13

**Authors:** B. A. Sabel, J. Wang, S. Fähse, L. Cárdenas-Morales, A. Antal

**Affiliations:** 1 Institute of Medical Psychology, Otto-von-Guericke University Magdeburg, Leipziger Strasse 44, 39120 Magdeburg, Germany; 2 Center for Behavioral Brain Sciences, Magdeburg, Germany; 3 Department of Clinical Neurophysiology, University Medical Center, Goettingen, Germany

**Keywords:** Vision restoration, Recovery, Low vision, Psychology, Stress, Personality, Predictive preventive personalized medicine, Glaucoma, Optic nerve damage, Psychology, Psychiatry, Ophthalmology, Neurodegeneration, Individualized patient profile, Neuroplasticity, Holistic medicine, Neuroticism, Vision rehabilitation

## Abstract

**Purpose:**

Identifying factors that affect recovery or restoration of neurological function is a key goal of rehabilitation in neurology and ophthalmology. One such factor can be prolonged mental stress, which may be not only the *consequence* of nervous system damage but also a major risk factor, or *cause*, of neural inactivation. Using the visual system as a model of neural injury, we wished to study how patients’ stress and personality profiles correlate with vision recovery as induced by therapy with alternating current stimulation (ACS) in patients with optic nerve damage.

**Methods:**

Personality and stress questionnaires were sent retrospectively to a clinical convenience sample of patients who suffer low vision due to optic nerve damage, which had previously been treated with ACS. The questionnaires included the NEO Five-Factor Inventory (NEO-FFI), the Trier Inventory of Chronic Stress (TICS), and the Flammer syndrome (FS) checklist, which probes signs of vascular dysregulation (VD). These scores were then correlated with the extent of ACS-induced vision restoration as recorded 1–3 years earlier by perimetric visual field tests.

**Results:**

Two NEO-FFI personality factors (lower neuroticism, higher conscientiousness) and the presence of physiological Flammer signs were associated with greater recovery as were individual items of the factors openness and agreeableness. Single NEO-FFI item analysis revealed that recovery relates to greater extraversion (optimistic and happy), openness (less guided by authorities for decisions on moral issues), and agreeableness (argue less, like working with others, thoughtful, considerate) as well as the presence of FS signs (cold hands/feet, hypotension, slim body shapes, tinnitus). This suggests that patients with better recovery were more calm, peaceful and secure, hard-working, and reliable, and with high organizational skills. In contrast, patients with poor recovery had a tendency to be emotionally unstable, anxious, unhappy and prone to negative emotions, impulsive, careless, and unorganized. Chronic stress assessed with TICS did not correlate with recovery.

**Conclusion:**

Vision restoration induced by ACS is greater in patients with less stress-prone personality traits and those who show signs of VD. Prospective studies are now needed to determine if personality has (i) a *causal* influence, i.e., patients with less stress-prone personalities and greater VD signs recover better, and/or (ii) if personality changes are an *effect* of the treatment, i.e., successful recovery induces personality changes. Though the *cause-effect* relationship is still open, we nevertheless propose that psychosocial factors and VD contribute to the highly variable outcome of vision restoration treatments in low vision rehabilitation. This has implications for preventive and personalized vision restoration and is of general value for our understanding of outcome variability in neuromodulation and neurological rehabilitation.

## Introduction

Low vision is one of the most feared diseases of the elderly, affecting more than 250 million people worldwide. Hence, finding means to improve or recover vision and identifying mechanisms of action are urgently needed.

Different pathologies of the central visual pathway can lead to vision loss due to damage of the retina, optic nerve, or brain. Because it is believed that functional impairment is mainly due to cell loss, the ensuing blindness was believed to be irreversible. However, contrary to this traditional paradigm, there is considerable recovery potential. Vision loss can not only spontaneoulsy recover to some degree [1, 2], but recovery can be initiated or potentiated by vision training [2–10] and non-invasive alternating current stimulation (ACS) [11–21]. For example, in a multicenter trial with patients suffering from optic nerve damage, transorbital ACS treatment resulted in 24% recovery of visual fields, measured by super-threshold perimetry. Despite these achievements, there is still considerable variability in outcome: while about one-third of patients do not respond much to vision restoration therapies, others experience moderate or massive improvements [12]. This treatment efficacy is similar across several independent vision restoration trials. Understanding the source (mechanisms) of this variability will enable us to design new, personalized treatment approaches in the field of regeneration, substitution, and restoration [13, 14].

When searching for possible sources of variability, we should consider the issue of neuronal activation after damage. According to our “theory of residual vision activation” [2], the visual system damage leaves behind regions of partial cell loss—called areas of residual vision (ARVs). They were proposed to provide a fundamental physiological basis of neurological recovery. Here, partially damaged tissue contains not only normally functioning and dead (or dying) nerve cells, but it also has inactive, *silent* neurons that suffer from a *hypometabolic* state. Such neurons are in a “locked-in” state; too healthy to die, but not healthy enough to fire nerve signals. According to our “neurovascular recovery hypothesis” [22], it is the re-activation of these silent neurons that might be a critical bottleneck for neurological recovery.

What is a potential cause of this hypometabolism? One possibility is endotheliopathy of local microvessels due to vascular dysregulation (VD), which reduces (but not stop) oxygen delivery [23–28] to levels which are sufficient for cell survival, but insufficient for sustained firing of action potentials. VD is a problem not only for the eye (e.g., in normal tension glaucoma [23, 24]) but also for the brain and other organs, leading to a cluster of symptoms and signs such as cold hands or feet, hypotension, diffuse visual field defects, and/or elevated oxidative stress, collectively described by the “Flammer syndrome” (FS) [29–35]. Furthermore, there are additional FS signs at different levels, including the hormonal (increased cortisol and endothelin-1), physiological (increased sensitivity to pain and drugs, lack of thirst, slim body shape), and psychological levels. Regarding the psychological level, it is interesting to note that FS patients have typical personality dispositions such as worrisome thinking, perfectionism, and ambitiousness (in sports and in their jobs), and they often have sleeping problems. These psychological aspects can be interpreted as signs of excessive mental stress, with or without patients being aware of them. We recently argued that stress is not only the *consequence* but also a possible *cause* of vision loss [36]. It may be that patients with FS are overly burdened by excessive and long-term stress, and/or have lower stress resilience. It is this experience of greater and often long-term stress in their lives, which starts a cascade of increased (long-term) stress hormone levels in the vascular system, endotheliopathy of the microvessels, impaired autoregulation (lack of timely vessel dilation), and subsequent neuronal inactivation (in the retina and brain) due to lack of sufficient levels of oxygen and glucose. If this causal chain of events is correct, then stress and personality may be the starting point of neuronal inactivation on the physiological level in the retina and/or brain with vision loss being the final result.

What’s more, the negative prognosis of progressive vision loss creates anxiety, which adds to the already existing stress level, initiating a vicious cycle of a downward spiral of progressive pathology. It is evident that people with newly diagnosed vision loss or a prognosis of inevitable progression inevitably experience stress, anxiety, fear, and/or depression [37–39]. Indeed, personality traits and scores of the visual function questionnaire (VFQ) correlate: patients with higher neuroticism or lower conscientiousness scores often reported lower scores in visual functional questionnaires [40]. Neuroticism and openness are essential predictors of general eye disease in adulthood, including glaucoma, diabetes-associated eye disease, cataract, etc. [41], and higher neuroticism and lower conscientiousness worsen the adaptability to visual loss [42, 43], which then reduces their ability to be able to cope with stress [44, 45]. Indeed, individuals experiencing adverse stress events had more peripheral stenosis and a slower central visual response [46–48].

These observations are compatible with our earlier proposal that stress may not only be the *consequence* of vision loss but also a possible *cause* (or risk factor) of losing vision: stress produces autonomic dysfunction, activates and later impairs the immune functions, elevates intraocular pressure, and hormonal and vascular dysregulation, with subsequent damage to the retina and optic nerve [36, 49]. Indeed, lowering stress through relaxation by way of meditation normalizes IOP, increases quality of life, and reduces stress-associated biomarkers with associated gene expression changes [50].

In summary, there is broad consensus that stress and personality have a profound influence on both the development of vision loss and how the patients succeed or fail to adapt to it. However, if and to what extent personality dispositions impact the recovery rate of vision is not yet known, i.e., whether the psychology of the patients can influence if they are responders or non-responders to vision restoration therapies.

Because there is a great need to learn more about psychosocial factors affecting vision recovery and restoration to clarify causes of response variability and move toward a more effective, personalized (and possibly preventive) treatment, we now explored how personality traits impact recovery of vision. To this end, we studied the influence of age, gender, personality traits, and chronic stress levels on outcome in patients who had previously been treated with repetitive transorbital ACS treatment, a vision restoration-inducing technique [for a recent review, see 51]. Specifically, our hypothesis was that personalities prone to stress would more likely suffer from VD and would most likely benefit from ACS, a technique known to improve blood flow. In addition, we wished to explore how demographic factors (age and gender) might influence vision recovery.

## Methods

We studied a clinical convenience sample of patients with optic nerve damage who suffered visual field defects and who had received ACS treatment about 1–3 years before filling out psycho-diagnostic questionnaires. These were sent to them by regular mail, and the psychological profile was then correlated with visual field recovery gains following ACS treatment. All subjects signed informed consent as mandated and approved by the Ethics Committee of the Otto-von-Guericke University School of Medicine in Magdeburg, Germany (approval no. 77/17).

### Participants and inclusion/exclusion criteria

**Fig. 1:**
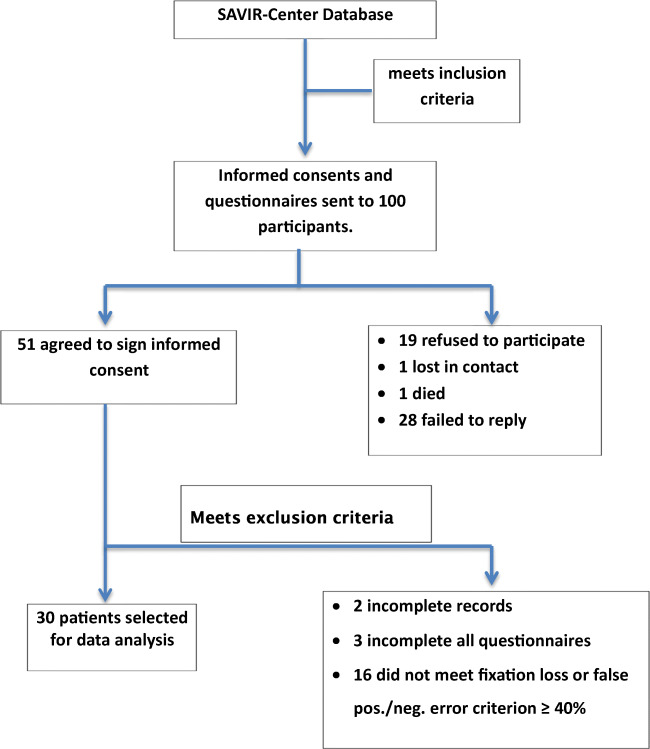
Participant recruitment

As a first step, a clinical convenience sample of the SAVIR-Center (www.savir-center.com) was screened for patients with visual field loss and who had previously been treated with ACS for 10 days (30–45 min each) in combination with psychological consulting during the period of 2015–2017. ACS was given with the aim of improving or restoring visual field function (for details, see [12]). A sample of 100 patients was contacted by mail with an invitation to fill out psychological questionnaires (see flowchart in Fig. 1). Fifty-one patients agreed to participate in the study, but only the data of 30 participants were considered for further analysis. They suffered from different diseases leading to visual field impairments including glaucoma (*n* = 15) and various other diseases, including posterior stroke, arterial retina occlusions, unexplained bilateral optic atrophy, embolization of left temporo-occipital meningioma, retina ablation, pilocytic astrocytoma, posttraumatic optic atrophy, recurrent hypophyseal adenomas, and retrobulbar neuritis.

We only included patients who (i) were 18 years or older; (ii) were able to complete the German language questionnaires; (iii) resided in Germany; and (iv) had monocular or binocular visual field defect caused by glaucoma or non-glaucomatous optic nerve damage. Considering that most of the patients are rather old, we chose the following exclusion criteria: (i) insufficient record of binocular vision test; (ii) fixation loss in Humphrey perimetry ≥ 40%; false-positive and false-negative error ≥ 40% (which is more liberal than the 30% international standard); (iii) prior history of epilepsy, photosensitivity, acute autoimmune diseases, mental illness diagnoses, or addictions; and (iv) complete vision loss in both eyes.

We reasoned that it would be justified that psychological questionnaires are filled out 1–3 years after patients received their therapy because personality is considered a relatively stable trait, and the passage of time posttreatment (within 3 years) should have only a minor influence on the results. This time gap, if anything, would increase variability, biasing the study results against our hypothesis and not in its favor.

### Questionnaires and visual field tests

A basic demographic questionnaire probed the age, gender, education level, working status, living conditions, and economic status. We selected physiological questions from the (non-standardized) FS checklist to count FS signs and symptoms which are believed to be associated with primary VD (courtesy of Dr. Flammer and Dr. Konieczka, University Eye Clinic Basel, Switzerland, 51) such as cold hands and cold feet, low blood pressure, and reduced thirst [24].

#### NEO Five-Factor Inventory

The NEO Five-Factor Inventory (NEO-FFI) probes five personality domains: extraversion, agreeableness, conscientiousness, neuroticism, and openness [52], and we used the validated German version [53]. *Extraversion* represents social behavior, assertiveness, and emotional expression. Here, a higher score represents an outgoing, warm, and adventure-seeking personality, whereas a lower score indicates a quiet, reserved, and withdrawn character. *Agreeableness* signifies cooperation, trustworthiness, and good-naturedness, with a higher score indicating helpfulness, trust, and empathy, and lower scores a critical, uncooperative, and skeptical personality. *Conscientiousness* is a trait reflecting competence, self-discipline, thoughtfulness, and goal-directedness. Here, a higher score is obtained by those who are hard-working and reliable, and with high organizational skills. A lower score suggests an impulsive, careless, and unorganized character. *Neuroticism* embodies the tendency toward unstable emotions, where higher scores are reached for traits such as anxiousness, unhappiness, and being prone to negative emotions and lower scores for calm, peaceful, and secure personalities. Finally, *openness* characterizes imagination, feelings, actions, and ideas. Here, a higher score is given for curiosity, broad interests, and independence, and lower scores for a practical and conventional personality that prefers routines.

#### Trier Inventory of Chronic Stress (TICS)

This test includes nine features: work overload, social overload, the pressure to perform, work discontentment, excessive demands at work, lack of social recognition, social tensions, social isolation, chronic worrying. It measures the individual experience of the most import chronic stressors in human life in terms of intensity, frequency, and duration [54]. Because the large majority of our patients are no longer in the workforce, we only analyzed non-work-related items.

#### Visual field tests

In contrast to the personality questionnaires, all vision tests were done before and immediately after completing the 10-day ACS treatment course. Humphrey field analyzer (HFA) (Carl Zeiss Meditec, Jena, Germany) and high-resolution perimetry (HRP) (NovaVision, Magdeburg, Germany) were used to measure the visual function of our patients. HFA is a widely used clinical perimeter that measures visual fields. It provides information about the location of any disease process or lesion in the entire visual pathway [55]. In contrast, HRP is a campimetric visual field test on a computer screen. Here, the visual fields represent blind regions (shown in black), areas of residual vision (shown in gray shading) revealing the extent of remaining vision, and intact visual field sectors (shown in white). Whenever a target stimulus was presented, or the fixation point changed its color, the individuals had to press a button. The target stimulus was a small, white dot which was presented randomly and twice in each test location. Fixation ability was quantified by measuring performance of the fixation point color change. Individuals unable to fixate properly frequently miss the color changes [3]. We recorded visual field index (VFI) and mean deviation (MD) values with HFA, and detective accuracy (DA), reaction time (RT), and correctly detected fixation controls (Fix) with HRP before and after treatment.

### Data analysis

An eye with a better VFI at baseline was defined as the “better eye.” Vision recovery was quantified by comparing pre- and post-treatment values (PRE–POST). Because some original data were already recorded as percentages, we used two methods to analyze vision recovery:

Absolute recovery = POST–PRE; Relative recovery = (POST–PRE) × 100/PRE. Because the MD is usually a negative value, we only analyzed its relative recovery. For the analysis of reaction time (RT), we inverted the number to facilitate interpretation such that greater recovery is represented by greater values as follows: absolute reduction = PRE–POST; relative reduction = (PRE–POST) × 100/ PRE. Given the small sample size, non-parametric Spearman correlation analyses were performed using IBM SPSS 24.0.

## Results

### Demographic and psychosocial characteristics

Demographic information of our 30 patients were as follows: gender ratio 17:13 (male:female); mean age 63.2 years (glaucoma 67 years, non-glaucoma 59 years); patients were highly educated, with 53.3% having attended college or equivalent. This is not surprising because the ACS treatment was private-pay; only few patients received health insurance reimbursement. Consequently, subjects did not worry about their financial situation because 96.7% of the patients had a stable income, and 40% were still working. In total, 86.7% participants lived with a partner or family member(s).

Patients filled out psychological questionnaires assessing personality traits (NEO-FFI), chronic stress (TICS), and Flammer signs with the FS checklist (Table 1). Most scores of the psychological questionnaires were within the normal range, i.e., with no sign of clinical psychopathology. The normal values for personality traits (*T*-score) and TICS (*T*-score) were found to be between 45 and 55. Thus, our sample had no bias in personality and chronic stress compared with the norm.

**Table 1:** Psychosocial factors

	All		Glaucoma		Non-glaucoma	
	Mean	SD	Mean	SD	Mean	SD
Personality traits (normal range = 45–55)						
Neuroticism	47.0	9.7	47.5	8.5	46.6	11.1
Extraversion	50.1	9.7	51.0	5.2	49.1	12.9
Openness	49.9	8.7	48.5	8.9	51.3	8.6
Agreeableness	53.9	11.3	52.8	12.0	55.1	10.8
Conscientiousness	53.0	10.6	49.7	7.6	56.4	12.3
Chronic stress (normal range = 45–55)						
Social overload	45.0	12.6	42.7	12.7	46.5	10.5
Pressure to perform	46.4	9.9	45.1	9.9	46.8	10.1
Lack of social recognition	50.2	10.3	48.7	9.9	51.1	10.9
Social tensions	48.5	9.2	51.5	8.1	45.9	9.7
Social isolation	50.6	9.7	54.6	6.7	45.4	9.9
Chronic worrying	49.9	8.4	51.5	8.2	49.0	8.6
Flammer syndrome (FS)						
Sum	12.7	4.9	13.3	5.7	12.1	4.1
No. of items with max FS signs (2 pts)	4	2.8	4.5	3.1	3.5	2.6
Total number of FS signs	8.7	2.7	8.9	3.2	8.6	2.1

The “FS checklist” is a non-standardized test, which contains 15 FS items with a maximum score for each item of 2 points (maximum possible total FS score = 30 points). In our sample, the mean total FS score was 12.7 points (range 4–23). We also counted the number of the FS items that our patients marked with the most severe symptom (2 points), hereafter referred to as severe FS signs. The average number of such items was 4, i.e., on average the patients marked 4 out of 15 items to be “severe FS signs.” Overall, the mean number of Flammer signs was 8.7 of a maximum of 15 points (range 3–13).

### Correlation among demographic features, psychosocial factors, and FS signs

We next studied the relationship between demographic and psychosocial factors, such as personality, chronic stress, and FS using Spearman correlation tests. As shown in Table 2, we found a negative correlation between age and social overload (*r* = − 0.374, *p* = 0.042), i.e., younger patients faced greater social pressure than older patients. Openness was positively correlated with social overload (*r* = 0.362, *p* = 0.049), and the frequency of severe FS signs (rating of 2 points) was positively correlated with age (*r* = 0.364, *p* = 0.048). This finding suggests that older participants suffered severe FS signs more frequently.

**Table 2:** Spearman correlation of psychosocial factors

		All			Glaucoma group					Non-glaucoma group				
		Age	Personality	FS	Personality		FS			Personality			FS	
			Openness	No. of items with max FS signs (2 pts)	Openness	Conscientiousness	Sum points	No. of items with max FS signs (2 pts)	Total number of present signs	Neuroticism	Extraversion	Agreeableness	Sum points	Total number of present signs
Age		/		0.364* (0.048)										
Chronic stress	Social overload	− 0.374* (0.042)	0.362* (0.049)			− 0.715** (0.003)MC	− 0.581* (0.023)	− 0.519* (0.047)	− 0.515* (0.050)					
	Pressure to perform					− 0.791** (0.001)MC				− 0.604* (0.017)		0.536* (0.039)		
	Lack of social recognition					− 0.666** (0.007)MC								
	Social tensions				0.573* (0.025)	− 0.541* (0.037)								
	Social isolation					− 0.715** (0.003)MC					− 0.612* (0.015)		− 0.723** (0.002)MC	− 0.684** (0.005)MC

*p < 0.05; **p < 0.01; *MC*, significant after Bonferroni correction when *p* < 0.0083

In the glaucoma group (*n* = 15), conscientiousness and FS points were negatively correlated with chronic stress, but openness was positively correlated with social tension. After Bonferroni correction, only conscientiousness remained significant. In the non-glaucoma group, neuroticism, extraversion, and FS points were negatively correlated with chronic stress, but agreeableness was positively correlated with pressure to perform. After Bonferroni correction, only the FS points were significant.

### Visual fields

Table 3 shows the change of visual function (recovery) using two calculation methods, the absolute and the relative change (percent change over baseline). As reported before, we found a great variability among patients, ranging from deterioration following ACS treatment to massive visual field improvement. Of note, with the exception of the mean deviation values, the average improvements were typically greatest in the worse eye.

**Table 3:** Vision recovery

	All						Glaucoma group						Non-glaucoma group					
	Absolute recovery			Relative recovery (% over baseline)			Absolute recovery			Relative recovery (% over baseline)			Absolute recovery			Relative recovery (% over baseline)		
	Median	Min	Max	Median	Min	Max	Median	Min	Max	Median	Min	Max	Median	Min	Max	Median	Min	Max
HFA																		
VFI better eye (%)	1.00	− 11.00	16.00	1.02	− 100.00	400.00	1.47	− 4.00	9.00	29.38	− 4.65	400	0.93	− 11.00	16.00	− 1.66	− 100.00	64.00
VFI worse eye (%)	1.50	− 52.00	60.00	4.17	− 99.00	900.00	7.13	− 5.00	60.00	76.32	− 35.71	900	− 1.80	52.00	8.00	13.23	− 99.00	200.00
MD better eye (dB)	0.43	− 3.42	2.56	/	/	/	0.27	− 3.42	2.56	/	/	/	− 2.71	− 45.74	1.24	/	/	/
MD worse eye (dB)	0.22	− 13.75	16.53	/	/	/	− 0.22	− 13.75	3.48	/	/	/	− 0.66	− 33.47	16.53	/	/	/
HRP																		
DA better eye (%)	0.53	− 20.47	11.64	0.63	− 59.47	15.48	1.98	− 10.21	11.64	3.36	− 10.92	15.48	− 1.22	− 20.47	4.93	− 4.24	− 59.47	9.06
DA worse eye (%)	3.24	− 10.04	16.16	7.22	− 40.68	59.97	3.64	− 10.04	10.74	10.72	− 11.57	59.97	1.92	− 9.23	16.16	3.15	− 40.68	49.78
RT better eye (ms)	0.00	− 45.00	147.00	0.00	− 9.18	20.65	19.00	− 45.00	147.00	2.89	− 9.18	20.65	0.53	− 37.00	56.00	− 0.29	− 9.05	10.31
RT worse eye (ms)	14.00	− 202.00	85.00	2.51	− 23.91	12.98	18.43	− 35.00	85.00	2.33	− 7.22	12.98	1.80	− 202.00	76.00	0.94	− 23.91	9.89
Fix better eye (%)	0.00	− 8.08	37.86	0.00	− 14.67	73.36	1.65	− 4.36	17.73	2.08	− 4.50	22.47	2.03	− 8.08	37.86	3.93	− 14.67	73.36
Fix worse eye (%)	1.03	− 15.40	42.18	1.10	− 18.52	79.02	0.75	− 6.08	26.14	1.56	− 6.79	37.65	6.16	− 15.40	42.18	11.73	− 18.52	79.02

*VFI*, visual field index; *MD*, mean deviation; *DA*, detective accuracy; *RT*, reaction time; *Fix*, fixation ability

### Correlation between psychosocial factors and vision recovery

**Fig. 2:**
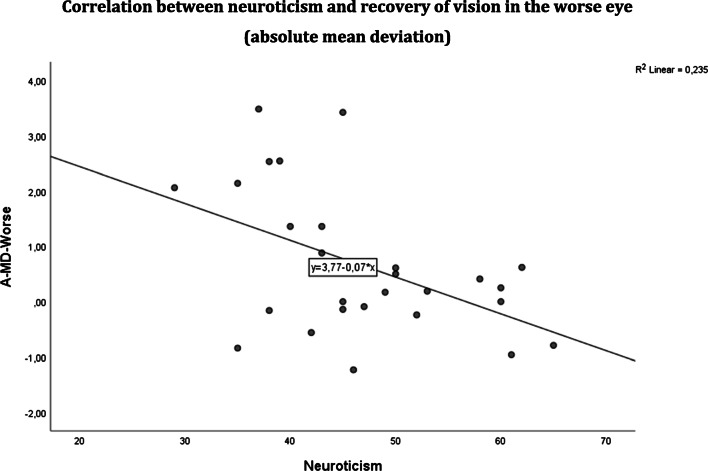
A-MD-Worse = recovery of absolute mean deviation in the worse eye (*p* = 0.005). Greater neuroticism was associated with less recovery

The main goal of our study was to study the relationship between psychosocial factors and vision recovery (Table 4). We found that higher neuroticism scores were associated with lower levels of absolute MD recovery in the worse eye (*r* = − 0.502, *p* = 0.005) (Fig. 2) and lower RT improvements in the better eye (*r* = − 0.365, *p* = 0.047). Furthermore, a higher FS sum score was positively correlated with absolute fixation recovery (*r* = 0.391, *p* = 0.036) in the worse eye as was the frequency of items with the maximum FS sign score of 2 points (*r* = 0.466, *p* = 0.011), i.e., patients with “severe FS signs” experienced better recovery.

**Table 4:** Spearman correlation of psychosocial factors with absolute vs. relative recovery of visual fields and reaction time

	All				Glaucoma group			Non-glaucoma group								
	Personality		FS		Personality	Chronic stress		Personality		Chronic stress					FS	
	Neuroticism	Conscientiousness	Sum points	No. of items with max FS signs (2 pts)	Neuroticism	Lack of social recognition	Chronic worrying	Openness	Conscientiousness	Social overload	Pressure to perform	Lack of social recognition	Social tensions	Chronic worrying	Sum points	No. of items with max FS signs (2 pts)
HFA																
A-VFI-Better												0.533* (0.041)				
A-VFI-Worse								− 0.554* (0.032)								
A-MD-Worse	− 0.502** (0.005)				− 0.718** (0.003)											
R-VFI-Worse					− 0.680* (0.011)			− 0.713* (0.006)								
HRP																
A-DA-Better						− 0.627* (0.012)	− 0.752* (0.001) MC				0.636* (0.011)		0.532* (0.041)			
A-DA-Worse						− 0.656* (0.011)						0.639* (0.010)				
A-Fix -Better									0.570* (0.027)							
A-Fix-Worse			0.391* (0.036)	0.466* (0.011)					0.559* (0.030)						0.606* (0.017)	0.605* (0.017)
A-RT-Better	− 0.365* (0.047)															
A-RT-Worse														− 0.520* (0.047)		
R-DA-Better						− 0.661* (0.007)	− 0.807* (0.000) MC			0.527* (0.043)	0.673* (0.006)		0.521* (0.047)			
R-DA-Worse										0.542* (0.037)		0.693** (0.004)				
R-Fix-Worse		0.420* (0.023)		0.474* (0.009)												0.653** (0.008)
R-RT-Worse														− 0.548* (0.035)		

*VFI*, visual field index; *MD*, mean deviation; *DA*, detective accuracy; *RT*, reaction time; *Fix*, fixation ability; *MC*, significant also after Bonferroni correction (critical *p* threshold: *p* < 0.0027); *A*, absolute; *R*, relative

*p < 0.05; **p < 0.01

In the glaucoma group, neuroticism, lack of social recognition, and chronic worrying were found to be negatively correlated with vision recovery, especially chronic worrying (which survived the Bonferroni correction). In the non-glaucoma group, openness and chronic worrying were negatively correlated with vision recovery, but conscientiousness, social overload, pressure to perform, lack of social recognition, social tensions, and FS were positively correlated with vision recovery.

Table 4 shows the relationship between psychosocial factors and relative visual field recovery. Here, conscientiousness was positively correlated with the relative fixation recovery in the worse eye (*r* = 0.420, *p* = 0.023).

### Correlation between single items and vision recovery

As we showed above, when calculating the correlations of vision recovery with total scores, only the factors “neuroticism” and “conscientiousness” were significantly correlated with vision recovery. However, to gain a better understanding of the patients’ psychosocial state, we also carried out a single-item analysis of all NEO-FFI and FS checklist items, i.e., correlating the score for each individual question with vision recovery (Table 5). Aside from the (expected) items in the domain of neuroticism and conscientiousness, several items in the other domains—extraversion, openness, and agreeableness—were also associated with vision recovery as follows:

- *Neuroticism*: Question 16 “I feel lonely or blue” was negatively correlated with absolute (*r* = − 0.471, *p* = 0.009) and relative (*r* = − 0.439, *p* = 0.015) DA recoveries in the better eye, negatively correlated with absolute reaction time (RT) reduction in the better eye (*r* = − 0.468, *p* = 0.009), and relative RT reduction in the better eye (*r* = − 0.451, *p* = 0.012); question 26 “Sometimes I feel completely worthless” was negatively correlated with absolute MD recovery in the worse eye (*r* = − 0.427, *p* = 0.019) and absolute RT reduction in the better eye (*r* = − 0.369, *p* = 0.045); question 41 “Too often, when things go wrong, I get discouraged and feel like giving up” was negatively correlated with absolute MD recovery in the worse eye (*r* = − 0.420, *p* = 0.021); and question 56 “At times I have been so ashamed I just want to hide” was negatively correlated with relative VFI recovery in the better eye (*r* = − 0.393, *p* = 0.031). These results suggest that patients had a greater chance for vision recovery, if they were proud of themselves, felt worthy and happy, or could always stay calm when facing troubles.
- *Extraversion*: Only question 12 “I consider myself especially “light-hearted” was positively correlated with absolute VFI recovery in the worse eye (*r* = 0.400, *p* = 0.029), i.e., patients had greater recovery if they were more optimistic and happy.
- *Openness*: Question 13 “I am intrigued by the patterns I find in art and nature” was positively correlated with relative recovery of fixation in the worse eye (*r* = 0.375, *p* = 0.045); question 38 “I believe we should not look to our religious authorities for decisions on moral issues” was positively correlated with absolute MD recovery in the better eye (*r* = 0.362, *p* = 0.049), and absolute (*r* = 0.404, *p* = 0.027) or relative (*r* = 0.441, *p* = 0.015) DA recoveries in the better eye. Thus, participants had better recovery if they were interested in art and nature and were more independent, i.e., less guided by authorities for decisions on moral issues.
- *Conscientiousness*: Question 20 “I try to perform all the tasks assigned to me conscientiously” was positively correlated with absolute (*r* = 0.367, *p* = 0.046) and relative (*r* = 0.367, *p* = 0.046) fixation recoveries in the better eye; question 40 “When I make a commitment, I can always be counted on to follow through” was positively correlated with absolute (*r* = 0.389, *p* = 0.037) and relative (*r* = 0.426, *p* = 0.021) fixation recoveries in the worse eye; question 45 “I’m as dependable or reliable as I should be” was negatively correlated with absolute VFI recovery in the worse eye (*r* = − 0.385, *p* = 0.032) but positively correlated with relative recovery of fixation in the worse eye (*r* = 0.408, *p* = 0.028); question 50 “I am a productive person who always gets the job done” was negatively correlated with absolute VFI recovery in the worse eye (*r* = − 0.638, *p* = 0.000); question 55 “I am able to get organized” was positively correlated with relative fixation recovery in the worse eye (*r* = 0.404, *p* = 0.030); and question 60 “I strive for excellence in everything I do” was positively correlated with relative fixation recovery in the worse eye (*r* = 0.388, *p* = 0.038). These results show that participants had a greater chance of vision recovery if they tried to perform all the tasks assigned to them conscientiously, or those who made promises that could be counted on; if they could organize themselves; strove for excellence in everything they did. But we also found that some factors related to the deterioration of vision, for example, people with higher scores in question 45 “I’m as dependable or reliable as I should be” and question 50 “I am a productive person who always gets the job done” had less absolute VFI recovery in the worse eye.
- *Agreeableness*: Question 9 “I do not often get into arguments with my family and co-workers” was positively correlated with relative VFI recovery in the better eye (*r* = 0.400, *p* = 0.029); question 19 “I would rather cooperate with others than compete with them” was positively correlated with absolute MD recovery in the worse eye (*r* = 0.433, *p* = 0.017); question 24 “I am not cynical and skeptical of others’ intentions” was negatively correlated with relative DA recovery in the worse eye (*r* = − 0.377, *p* = 0.044); question 34 “Most people I know like me” was negatively correlated with relative DA recovery in the worse eye (*r* = − 0.370, *p* = 0.048); and question 49 “I generally try to be thoughtful and considerate” was positively correlated with absolute MD recovery in the worse eye (*r* = 0.395, *p* = 0.031). These results indicate that participants had better vision recovery if they were not easily arguing with their family and colleagues, liked to work with others, and generally tried to be thoughtful and considerate. At the same time, we also found that some factors are related to the deterioration of vision such as a higher score of question 24 “I am not cynical and skeptical of others’ intentions” and 34 “Most people I know like me” with less relative DA recovery in the worse eye.

**Table 5:** Spearman correlation between vision recovery and personality traits/Flammer syndrome signs

	Neuroticism-				Extr+	Openness+		Conscientiousness+						Agreeableness+					Flammer syndrome+			
	16	26	41	56	12	13	38	20	40	45	50	55	60	9	19	24	34	49	2	3	12	14
HFA																						
Absolute VFI better eye																						*0.39* *
Absolute VFI worse eye					*0.40* *					− 0.39 *	− 0.64 **MC											
Relative VFI better eye				*− 0.40* *										0.40*								
Relative VFI worse eye																					0.47*	
Absolute MD better eye							*0.36* *															
Absolute MD worse eye		*− 0.43* *	*− 0.42* *												0.43*			*0.40* *				
HRP																						
Absolute DA better eye	*− 0.47* **						*0.40* *															
Relative DA better eye	*−0.44* *						*0.44* *															
Relative DA worse eye																− 0.38 *	− 0.37 *					
Absolute Fix better eye								*0.37* *														
Absolute Fix worse eye									*0.39* *										*0.38* *	*0.37* *	*0.44* *	
Relative Fix better eye								*0.37* *														
Relative Fix worse eye						*0.38* *			*0.43* *	*0.41* *		*0.40* *	*0.39* *						*0.37* *		*0.44* *	
Absolute RT better eye	*− 0.47* **	*− 0.37* *																				− 0.39 *
Relative RT better eye	*− 0.45* **																					− 0.40 *

Correlation between vision recovery and NEO-FFI personality single-item scores and Flammer signs. *VFI*, visual field index; *MD*, mean deviation; *DA*, detective accuracy; *RT*, reaction time; *Fix*, fixation controls. *MC*, after Bonferroni correction (*p* < 0.0027). NEO-FFI items of the factors neuroticism, extraversion, openness, conscientiousness; agreeableness and with the Flammer syndrome signs; The + and - signs for the NEO-FFI personality factors represent the level of stress burden (- more, + less stress). If the direction of the correlation matches the respective personality factor, this is considered “congruent” with the “stress personality” hypothesis. As shown in italics, 32/36 items were congruent, i.e., patients with a personality with less stress burden showed greater recovery. *p < 0.05; **p < 0.01. Of note, the only incongruent correlations were found in conscientiousness and FS signs

A single-item analysis was also carried out for the FS checklist. As shown in Table 5, the FS question 2 “Do you feel cold when you sit down quietly for some time or when you are not moving?” was positively correlated with absolute (*r* = 0.381, *p* = 0.041) and relative (*r* = 0.374, *p* = 0.046) fixation recoveries in the worse eye; question 3 “Do you have or have you ever had a low blood pressure?” was positively correlated with absolute fixation recovery in the worse eye (*r* = 0.373, *p* = 0.046); question 12 “body shape at the age of 20-30 years” was positively correlated with relative VFI recovery in the worse eye (*r* = 0.471, *p* = 0.015), and absolute (*r* = 0.435, *p* = 0.018) and relative (*r* = 0.435, p = 0.018) fixation recoveries in the worse eye; and question 14 “Have you had phases in your life in which you had ringing in your ear (tinnitus)?” was positively correlated with absolute VFI recovery in the better eye (*r* = 0.386, *p* = 0.035), and negatively correlated with absolute (*r* = − 0.386, *p* = 0.035) and relative (*r* = − 0.400, *p* = 0.028) RT reduction in the better; i.e., the tinnitus sign had mixed results. These results showed that patients with some of the severe FS signs (feeling easily chilly, hypotension, slim body shape) had greater chances of vision recovery.

To interpret the single-item analysis, we assumed that persons with a high level of stress burden are those with high scores on neuroticism and low scores on all other personality factors: lower conscientiousness, lower agreeableness, and lower openness, and a slight tendency toward more introversion (the “stress personality” hypothesis).

Using this assumption, we classified each significant correlation value as being either congruent or not congruent with this hypothesis. In Table 5, the positive or negative direction of the correlation shows if each correlation coefficient is congruent or not congruent with the stress personality hypothesis. Indeed, 32/36 items were congruent, which is a rather consistent correlation pattern. It confirms the hypothesis that personality, stress, and recovery are related. Moreover, we calculated the two groups separately and found more significances with item congruence of 40/56 in the glaucoma and 51/70 in the non-glaucoma groups.

These results suggest that patients recover better if they have less stress-prone personalities. Though our stress questionnaire did not reveal any correlations between reported stress and vision recovery, persons with personality traits that create greater stress levels throughout their lifetime recovered poorly. Such patients tend to have higher levels of neuroticisms, lower conscientiousness, lower agreeableness, and lower openness, and a slight tendency toward more introversion (“stress personalities”).

It may be argued that “stress personality” is unrelated to the personality profile as assessed by the NEO-FFI. To test this possibility, we carried out a single NEO-FFI item analysis to check if the direction of their correlation with recovery matches the stress personality profile. To this end, we classified each significant correlation according to one of four options in a 2 × 2 factor matrix: positive vs. negative correlation (factor A) and “high stress personality” yes vs. no (factor B). Chi-square analysis revealed that 23 of the 27 such correlations were compliant with our hypothesis (chi-square test (1, *n* = 27), *χ*^2^ = 5.87, *p* < 0.05). This was confirmed by a separate chi-square analysis for the two sub-groups which had almost twice as many significant correlations. In the glaucoma sub-group, 37/50 were compliant with our hypothesis (chi-square test (1, *n* = 50), *χ*^2^ = 11.71, *p* < 0.05) and in the non-glaucoma sub-group 43/54 (chi-square test (1, *n* = 54), *χ*^2^ = 10.96, *p* < 0.05). Thus, the single NEO-FFI item analysis confirms the stress personality hypothesis even more clearly.

In summary, the patients with the greatest chance of recovery from visual system damage (following ACS treatment) are those with more FS signs and those with lower stress personality (Table 6 shows the FS checklist items translated to English by the authors).

**Table 6:** Flammer syndrome checklist

1. Do you suffer from cold hands or feet (possibly also in the summer) or have other people ever told you that your hands are cold?	
2. Do you feel cold when you sit down quietly for some time or when you are not moving?	
3. Do you have or have you ever had a low blood pressure?	
4. Do you ever feel dizzy when you suddenly stand up from a lying down (or resting) position?	
5. Do you need a relatively long time to fall asleep (e.g., when you are cold)?	
6. How is your feeling of thirst?	
7. How often do you have a headache?	
8. In case you suffer from migraines, do you have accompanying symptoms (e.g., visual disturbances, transient altered sensation [e.g., itching] in your arms or in your legs etc.?)	
9. If you have to take medications (other than painkillers), do you have the feeling that you react strongly to them and/or that you would feel better if you would take a lower dose than that which is normally prescribed?	
10. Do you suffer from any type of pain (for which you would have to take pain killers)?	
11. How well can you smell? Can you smell things that other people do not smell or that others smell to a lesser extent?	
12. Please mark the following: At 20–30 years of age, I was …(slim, normal, overweight)	
13. If you had to judge yourself (e.g., in your work), would you say that you are particularly reliable with a tendency towards perfectionism?	
14. Have you had phases in your life in which you had ringing in your ear (tinnitus)?	
15. Have you noticed reversible blotches (white or red) on your skin when you were excited or angry (e.g., during stress)?	

Reprinted with the courtesy of Prof. Josef Flammer, University of Basel Eye Hospital, Switzerland

## Discussion

Stress and personality are not only known to contribute to the progression of visual pathology, but they also influence patients’ ability to adapt to vision loss and quality of life [36]. Though our clinical experience is that psychosocial factors influence the extent of recovery, i.e., if a given patient will be a responder or non-responder, there is no empirical study of this potential relationship. Though the number of reports on vision restoration by different therapies (e.g., vision restoration training, ACS) is steadily increasing, their outcome is still highly variable. We urgently need to learn more about the role of physiological, psychological, and demographic factors that contribute to recovery of neurological functions in general and recovery of vision specifically. In the present study, we therefore analyzed the demographic and psychosocial profiles of a small convenience sample of patients suffering from vision loss, in which vision recovery was induced by ACS, a method known to improve visual fields [11, 12]. As we report here, age and gender did not affect vision recovery, while psychosocial factors (personality profiles) and physiological signs of VD had a profound influence on vision recovery. Specifically, “low stress personality” traits, i.e., lower neuroticism and greater conscientiousness, were positively correlated with vision recovery after ACS treatment as did some individual items of greater agreeableness and greater openness. In addition, we found that greater recovery was observed in patients with physiological signs of VD, a hallmark of FS [30].

In contrast to our original hypothesis, our correlations suggest that “stress-prone” personalities benefited (recovered) less from ACS and those with Flammer signs recovered more. Surprisingly, stress questionnaire profiles did not correlate at all with vision recovery. This may be due to a notable limitation of our study, namely, that our questionnaires were filled out by the patients long after the therapy had ended. Therefore, we cannot be certain about the *cause-effect* relationship. Though personality is considered a rather stable trait, vision loss, known to induce stress and reduce quality of life, may also have a profound influence on a person’s response in psychological questionnaires, which were collected several months or years *after* the therapy. Therefore, the following two possibilities exist: (i) either patients with a stress-prone personality respond less well to the therapy, or (ii) those who profited most from the therapy scored less neurotic and more conscientious as a result of a more optimistic outlook on life. In more simplified words, stress can either prevent recovery, or the experience of recovery leads to a fundamental personality change characterized by less stress, and a more optimistic, less neurotic, and more conscientious mental state. Which of these two possibilities is true, i.e., the nature of the cause-effect relationship, can only be answered in future prospective studies.

However, it is interesting to note that patients with FS signs of VD experienced greater recovery. Hence, this finding is compatible with the hypothesis that ACS, known to improve blood flow [21, 56], improves vision by way of normalizing blood flow. If this is confirmed by prospective studies, this would support our earlier proposal that neurological recovery is—at least in part—due to the normalization of blood flow. In this case, VD may be both a problem and a solution for neurological dysfunction: impaired autoregulation is a cause of neuronal inactivation and functional loss, and improvement of autoregulation is a major underpinning for recovery [22].

While our retrospective study should be interpreted with caution, our results support the notion that psychosocial factors and stress play an important role in neurological recovery. This is compatible with prior studies.

### Personality, stress, and restoration of vision

Clinical experience and prior studies already suggest that mental stress is one of the main causes of vision loss, and a patient’s personality might lead to—or be affected by—stress. This was reported, for example, by Fontana [57] who found a significant negative correlation between stress and extraversion. They used the Professional Life Stress Scale (PLSS) to assess teachers’ stress levels and correlated it with personality dimensions as measured with the Eysenck Personality Questionnaire (EPQ). They observed significant positive correlations between stress and high scores of psychoticism and neuroticism, with neuroticism being the best predictor of stress levels, with age or gender having little influence. This supports our assumption that in low vision patients stress and personality traits are related.

### Personality and vision recovery

It is conceivable that personality can have two different influences on visual performance and recovery: On one hand, it may have an influence on the actual performance during a testing session (state), or, on the other hand, it can be a stable, physiologically relevant influence of overall mental stress and stress hormone levels, which may last over months and years, impairing blood flow and neural function (trait).

In our study, we identified at least two out of five personality traits (neuroticisms, conscientiousness) to be significantly correlated with vision recovery. Specifically, patients who benefited from the ACS therapy were calmer, more peaceful and safer, more diligent, reliable, and organized in their lives. In addition, a single-item correlation of two other personality traits (openness, agreeableness) confirmed the overall finding.

#### Neuroticism

Regarding the personality factor *neuroticism*, we found a significantly negative correlation with recovery. Neuroticism was studied in low vision patients also by Gaynes et al. [58] who evaluated if this personality trait is associated with decreased ability to adapt to change and that this modifies the association of vision impairment and cognition. Visually impaired subjects with high neuroticism had also a lower cognitive score than those with a low neuroticism level. Others [59–61] found that neuroticism was associated with decreased attentional control over the visual field, and it was proposed that neuroticism decreased attentional disengagement. We showed that lower neuroticism correlated significantly with vision recovery, but a prospective study with a larger sample should establish the cause-effect relationship.

#### Conscientiousness

High scores in this trait characterize persons who are self-disciplined, thoughtful, and goal-driven. It also indicates effort and super-ordinate control [60] which, in turn, affects social performance, such as academic achievement [61] and work engagement [62]. Individuals with high scores in conscientiousness are those more concerned with tasks and goals in the face of irrelevant information [60]. We found that higher scores positively correlated with vision recovery.

### FS and vision recovery

Besides having demonstrated the correlation of personality and recovery, we also assessed how absolute and relative recoveries of visual functions relate to the severity of FS signs. We found that those patients who had severe symptoms of VD on the FS checklist recovered well. According to the theory proposed by Flammer, the higher the FS checklist value, the more likely will patients suffer from VD, which is one of the main causes of vision loss (the other being increased intraocular pressure). Indeed, glaucoma is known to be associated with hemodynamic changes and reduced blood flow regulation in the blood vessels [23, 63]. We have recently argued that better blood flow (autoregulation) leads to better recovery [22]. The reason is that different therapies can activate (improve) residual vision including vision restoration training, non-invasive brain stimulation, or blood flow–enhancing medications. Proposed mechanisms include the reorganization of brain functional networks and improved vascular regulation, both of which support recovery and restoration. Considering that ACS enhances blood flow, our observation of greater recovery in patients with FS signs supports the proposal that patients affected by VD also benefit more from blood flow–enhancing ACS.

We have recently suggested that a major cause of the FS is mental stress [36], which now raises the question how stress and vision restoration relate.

### Chronic stress and vision recovery

It is known that severe chronic worrying and VD are related to stress [36], increasing the risk of coronary heart disease [64] and glaucoma, due to hemodynamic changes in the eye and other organs [23, 63]. Surprisingly, in the present study, we found no correlation between the level of chronic stress and the magnitude of vision recovery. Therefore, in our patient group, a direct link of Flammer signs, personality disposition, and mental stress could not be established. Several reasons might explain this missing correlation: (i) the sample size of our study was too small; (ii) the TICS questionnaire is not sensitive enough or inappropriate because it contains many items related to the working age group to which only 40% of our patients belonged; (iii) the retrospective study design with its inherent ambiguity regarding the *cause-effect* problem is insensitive to answering this question; and (iv) persons unable to recognize their stress-associated feeling and actions (“I am not stressed at all”) might be more prone to psychosomatic reactions. This might lead to a mismatch between subjective perception of stress and how the body responds to stress.

### Correlating individual items of the NEO-FFI and FS checklist with vision recovery

Because only the factors of *neuroticism* and *conscientiousness* correlated significantly with vision recovery, we next wished to explore the role of the other three personality traits as well (agreeableness, openness, and extraversion). As shown in Table 5, a number of individual items from these domains correlated with vision recovery. The direction of most of the correlations was consistent with the hypothesis that stress personality is negatively correlated with recovery as confirmed by the chi-square tests.

### Comparing vision recovery glaucoma and non-glaucoma patients

When dividing the results of our patients into two groups with or without glaucoma, we found that the correlation of personality traits and vision recovery was somewhat different. Whereas in glaucoma patients poor recovery was associated with neuroticism, lack of social recognition, and chronic worrying, in non-glaucoma patients poor recovery was also associated with chronic worrying and lack of social recognition. But these patients had greater openness, conscientiousness, social overload, pressure to perform, and FS signs.

Because the non-glaucoma group contains many different diseases, it is difficult to explain the exact influencing factor. Yet, even this subdivision of groups confirms that neuroticism, lack of social recognition, and chronic stress play a very important role.

While our retrospective study does not inform us about causality, we assume that personality traits are rather stable, i.e., patients’ personality is similar before therapy and several years after therapy. In the Yan study [65], the authors found that glaucoma patients had higher scores of neuroticism, and, with logistic regression analysis, Cheng [41] made similar observations that neuroticism was one of the significant predictors of self-reported eye condition. We therefore propose that neuroticism affects glaucoma patients not only before but also after the treatment.

Lack of social recognition and chronic worrying were two other factors that affected our results. The present study generally confirms our early literature review showing that mental stress affected vision loss, and the study also showed that mental stress affected vision recovery in the glaucoma [36].

We speculate the following: (personality-mediated) chronic stress and worrying are a major cause of vision loss. Possibly, by way of inducing VD in the eye and brain, such stress and worrying can inactivate neurons. Therefore, patients with VD benefit most from the blood flow–enhancing ACS therapy.

### Other sources of variability

There are also other sources of variability. One source is age, which correlates with the reduction of the visual fields [66] and also correlates with improvement by using vision restoration therapy (VRT). For example, a large clinical observational study showed that VRT improved vision by 17.2% and those over the age of 65 benefited most [10].

There are also gender differences in blindness around the world; about two-thirds of those affected are female, and the most impoverished women are particularly vulnerable to life-threatening limitations due to visual impairment [67]. Also, of all people affected by FS, about 70% are women, who have a greater tendency to suppress anger because of stereotypic feminine gender socialization. This phenomenon was attributed to the traditional women’s role found in such patients [68].

## Methodological limitations and suggestions for future studies

Our research has several limitations: (i) our clinical convenience sample is not representative of the general population as we studied private-pay patients who tend to be more wealthy, more flexible to try new solutions, more curious about new therapies, and willing to take risks (spending time and money) and to face challenges. (ii) Most of our patients were elderly and the causes of the diseases were rather variable; (iii) our sample size was small, reducing the power of our tests, thus providing only exploratory insights regarding the role of psychosocial factors in recovery; (iv) the retrospective study design does not inform us about the *cause-effect* relationship, i.e., if a stressed personality prevents recovery or if recovery modified the personality in such a way that the patients became less neurotic and more conscientious, open, and agreeable; and finally, (v) the FS checklist is not yet a validated questionnaire that satisfies test quality criteria.

Furthermore, no correlation was found between the stress questionnaire and vision recovery. Reasons could be (i) the small study sample, and (ii) the fact that our stress questionnaire included many work-related items, but most of our patients no longer worked.

Yet, there is a “chicken-egg” problem of not knowing what the cause and what the effect. While we assume that personality dispositions are stable over time, irrespective of which treatment the patients receive, we cannot exclude the reverse possibility, i.e., that those patients with excellent vision recovery responded differently to psychological questionnaires, which were sent to them several years later. For example, patients with excellent vision recovery may have become less neurotic and more calm and conscientious, i.e., their personality changed. Despite these limitations, our study provides valuable hints how psychosocial factors might affect, or be affected by, neurological recovery of low vision. To overcome these limitations, a prospective study with a larger sample size is needed where validated questionnaires are filled out before treatment starts. Only then can we say more about the cause-effect relationship between personality, stress, FS, and vision recovery.

## Conclusion

Age and gender have apparently little or no influence on vision recovery, which is in agreement with previous observations. But—as we show here—personality dispositions correlate with vision restoration; namely, those with higher FS scores, lower neuroticism, and higher conscientiousness had greater recovery while those who chronically worry had less recovery. Our findings suggest that while ACS therapy is useful and the worse eye benefits more, psychosocial factors have a great impact on the magnitude of recovery.

When considering both the average results and the single-item analysis, these are the characteristics of the “ideal” personality with the best recovery potential:

(i) Tendency toward *extraversion*, i.e., outgoing, warm personality, adventure-seeking;
(ii) High on a*greeableness*, being cooperative, trustworthy, and good-natured, with greater helpfulness and trust;
(iii) Greater level of *conscientiousness* with competence, self-discipline, thoughtfulness, and goal-directedness, reliability, and good organizational skills;
(iv) Lower level of *neuroticism*, i.e., being calm, peaceful, and secure;
(v) Greater *openness* characterized by more imagination, feelings, actions, and ideas with greater curiosity, broad interests, and independence.

**Fig. 3:**
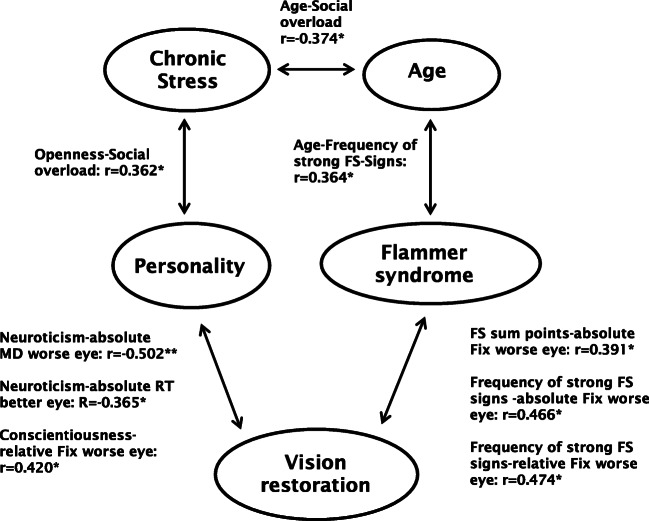
Relationship of vision recovery and psychological and demographic factors

Though the lack of a prospective study limits the validity of our conclusions, less recovery is expected in patients with high scores of *neuroticism* (unstable emotions, anxious, unhappy, prone to negative emotions) and low scores in *agreeableness* (critical, uncooperative, and skeptical personality), *conscientiousness* (impulsive, careless, and unorganized), and *openness* (practical and conventional personality, preferring routines) (Fig. 3).

Further research is now needed to determine the cause-effect relationship, i.e., if greater vision recovery induces a personality change or if a (stable, calm) personality disposition has a positive influence on how responsive the body (the eye, brain, vascular system) is to treatment, i.e., recovery of vision. We believe there are several reasons why certain psychosocial predispositions are the *cause*, not the *effect*, of better recovery: (i) personalities are considered to be rather stable across a lifetime; (ii) excessive, long-term mental stress, which is personality-dependent, is perhaps the main cause of VD which, in turn, is the pathological underpinning of glaucoma and neural dysfunction (inactivation) [22]; and (iii) while improved vision increases quality of life in low vision patients, we believe that a 10-day ACS treatment course, known to improve blood flow [21, 32], is not sufficiently powerful to change a person’s fundamental personality disposition, reducing neuroticism and increasing conscientiousness.

Of course, in reality, most patients are not on one or the other end of the personality extremes. And there are other factors that impact recovery rates such as the magnitude of the vision loss (cell number, their activation state), the nature of the visual field loss (defect depth, size of relative defect) [69], lifestyle (sports, nutrition, drug/alcohol abuse), dehydration, and concomitant medical conditions. But, as we now showed for the first time, psychosocial factors (personality) are a major (and measurable) source of variability, explaining why some patients recover better and others do not. If our proposal is confirmed by prospective studies, it would mandate a holistic treatment approach combining vision restoration therapy—by whatever means (ACS, vision training)—with psychological intervention to reduce stress and mental burden, including relaxation techniques (such as meditation) [50, 70]. By addressing different mechanisms in the eye-brain-vascular triad [22], we might be able to reduce variability and increase vision restoration outcome.

In summary, we conclude that vision restoration influences, or is influenced by, psychosocial factors (personality traits) and VD. Prospective studies are now needed to determine if personality has (i) a *causal* influence, i.e., patients with less stress-prone personalities and greater VD recover better; and/or (ii) if personality changes are an *effect*, i.e., being induced by recovery. From clinical experience, our best guess would be that both are true. Whatever the *cause-effect* relationship may be, we propose that psychosocial factors and VD contribute to the highly variable outcome of vision restoration treatments in low vision rehabilitation.

## Expert recommendations

Given that psychological factors are critical for recovery of vision, several recommendations can be made which might also apply to neurological rehabilitation in general. (i) Studies, which evaluate efficacy of neurological or ophthalmological recovery, rehabilitation, and restoration, should consider personality as a critical co-variable. (ii) Stress reduction (such as meditation) is an important adjuvant to improve outcome in neurological and ophthalmological rehabilitation. Finally, (iii) patients should learn ways to reduce their mental stress not only during but also after therapy because it could slow the progression of disease and/or enhance further recovery potentials. In sum, personality and stress are important elements to consider for personalized and preventive neurorehabilitation and neuromodulation.
